# Evaluation of Cerebral White Matter in Prelingually Deaf Children Using Diffusion Tensor Imaging

**DOI:** 10.1155/2018/6795397

**Published:** 2018-02-04

**Authors:** Kye Hoon Park, Won-Ho Chung, Hunki Kwon, Jong-Min Lee

**Affiliations:** ^1^Department of Otorhinolaryngology-Head and Neck Surgery, Soonchunhyang University College of Medicine, Cheonan, Republic of Korea; ^2^Department of Otorhinolaryngology-Head and Neck Surgery, Samsung Medical Center, Sungkyunkwan University School of Medicine, Seoul, Republic of Korea; ^3^Department of Neurology, Yale University School of Medicine, New Haven, CT, USA; ^4^Department of Biomedical Engineering, Hanyang University, Seoul, Republic of Korea

## Abstract

This study compared white matter development in prelingually deaf and normal-hearing children using a tract-based spatial statistics (TBSS) method. Diffusion tensor imaging (DTI) was performed in 21 prelingually deaf (DEAF group) and 20 normal-hearing (HEAR group) subjects aged from 1.7 to 7.7 years. Using TBSS, we evaluated the regions of significant difference in fractional anisotropy (FA) between the groups. Correlations between FA values and age in each group were also analyzed using voxel-wise correlation analyses on the TBSS skeleton. Lower FA values of the white matter tract of Heschl's gyrus, the inferior frontooccipital fasciculus, the uncinate fasciculus, the superior longitudinal fasciculus, and the forceps major were evident in the DEAF group compared with those in the HEAR group below 4 years of age, while the difference was not significant in older subjects. We also found that age-related development of the white matter tracts may continue until 8 years of age in deaf children. These results imply that development of the cerebral white matter tracts is delayed in prelingually deaf children.

## 1. Introduction

Cochlear implantation (CI) is the only rehabilitative method that restores auditory sensation in profoundly deaf subjects, but improvements in hearing and speech following successful CI are inconsistent, especially in prelingually deaf subjects [1]. Many researchers have reported on the critical factors that determine the success of auditory language rehabilitation following CI, and the results suggest that the younger deaf children are at the time of CI, the better the outcome is. Consequently, CI surgery is more likely to be successful when performed on younger deaf children than on older ones [2]. However, several traditional demographic factors, including age at implantation, duration of deafness, number of inserted electrodes, and mode of communication, explained less than 50% of the observed variance in outcomes among implanted children [3]. Biological factors have also been investigated, such as the relationship between residual spiral ganglion cell populations in the temporal bones of CI patients and their speech perception ability during life [4]. However, an unexpected negative correlation suggested that certain processes in the central nervous system (CNS) are at least as important as peripheral factors.

A great deal of evidence supports the development of plastic changes in the brains of congenitally deaf subjects [5]. In a signing deaf subject, some processing of visual information is performed in the auditory cortex [6], indicating that the brain copes, to some extent, with alterations in sensory input. This is one reason why hearing after CI seems to be poor in prelingually signing deaf adults [1]. One study using positron emission tomography (PET) reported that the under-used auditory cortices of prelingually deaf individuals gradually changed over time, being initially hypometabolic and later normal or hyperactive [7]. An association was also evident between better perception after CI and more pronounced presurgical hypometabolism in the superior temporal regions. Removal of sensory receptors in young animals exerted profound effects on the maturation of brain stem neuronal structures [8]. One example of such changes is sensory deprivation-induced cell death, evident in a variety of models when afferent activity is interrupted during development [9, 10]. The poorly understood phenomenon of deprivation-induced cell death and other experience-induced changes in neural structure and function, that is, alterations in normal activity patterns during finite periods early in life, dramatically change the CNS, whereas identical manipulations later in life exert little or no effect [11]. We hypothesized that a similar phenomenon might occur in congenitally deaf children, associated with a delay in development or myelination of cerebral white matter. However, white matter development has not yet been evaluated in prelingually deaf children. Our purpose here was to explore differences in cerebral white matter development in prelingually deaf children compared with that in normal children via diffusion tensor imaging (DTI).

## 2. Materials and Methods

### 2.1. Subjects

Twenty-one profoundly hearing-impaired children (8 boys and 13 girls; mean age: 3.9 (range: 1.7–7.7) years; DEAF group) were included. DTI was performed when conventional MRI was taken as a presurgical evaluation for CI. The control group included 20 normal-hearing children (12 boys and 8 girls; mean age: 4.7 (range: 2.0–7.6) years; HEAR group). Cerebral anatomy, as depicted by conventional MRI, was normal; none of the 41 children had any clinical history of neurological disease or developmental abnormality. The study was approved by our institutional review board. Written informed consent was obtained from all parents. All children were sedated with chloral hydrate administered by a radiologist prior to MRI.

### 2.2. Image Acquisition

All scans were acquired using a Philips 3.0 T scanner (Philips Achieva, Philips Medical System; Best, Netherlands). DTI was obtained via single-shot echo-planar acquisition from 45 noncollinear, noncoplanar diffusion-encoded gradient directions with the following parameters: *b*-value = 1000 s/mm^2^, TR/TE = 3700 ms/80 ms, matrix = 128 × 128, slice thickness = 3 mm, and FOV = 180 × 180 mm^2^. Two images with no diffusion weighting (*b*-value = 0 s/mm^2^) and diffusion weighting (*b*-value = 1000 s/mm^2^) were acquired for each slice and each gradient direction.

### 2.3. DTI Preprocessing

DTI data were processed using FMRIB Software Library (FSL) (http://www.fmrib.ox.ac.uk/fsl). Motion artifacts and eddy current distortions were corrected by normalizing each directional volume to the non-diffusion-weighted image (*b*0) using the FMRIB Linear Image Registration Tool (FLIRT) with 6 degrees of freedom. After correcting motion artifacts and eddy current distortions, the diffusion tensor was calculated, normally using a simple least squares fit of the tensor model to the diffusion data to calculate the three eigenvalues (*λi*, *i* = 1,2, 3) and eigenvectors (*εi*, *i* = 1,2, 3). Then, the fractional anisotropy (FA), an index of directional selectivity of water diffusion, and the mean diffusivity (MD) were determined for every voxel according to standard methods using the program DTIFIT in FSL.

### 2.4. Tract-Based Spatial Statistics (TBSS) Analysis

The fractional anisotropy (FA) images of the DTI preprocessing result were used in the TBSS analysis [12, 13]. In adult studies, all FA images are aligned onto a standard FMRIB58 FA template provided with FSL using a nonlinear registration algorithm implemented in the TBSS package. However, this approach is not appropriate for children's data. A new target image was selected: the one with the minimum mean displacement score from all other subjects in the group using TBSS option (tbss_2_reg -n) [12]. The original FA images were transformed to the new target image using linear and nonlinear registration methods. The transformed FA maps were averaged to make a group-specific template, which was inserted in the standard TBSS protocol. All of the original FA images were aligned onto the group-specific template using linear and nonlinear registration methods. To create a skeletonized mean FA image, the FA images that aligned on the group-specific template were averaged. Each subject's (aligned) FA image projects onto the skeleton by filling the skeleton with the highest FA values from the nearest relevant center of fiber tracts. A threshold FA value of 0.2 was chosen to exclude voxels of adjacent grey matter or cerebrospinal fluid.

### 2.5. Statistical Analysis

**Figure 1:**
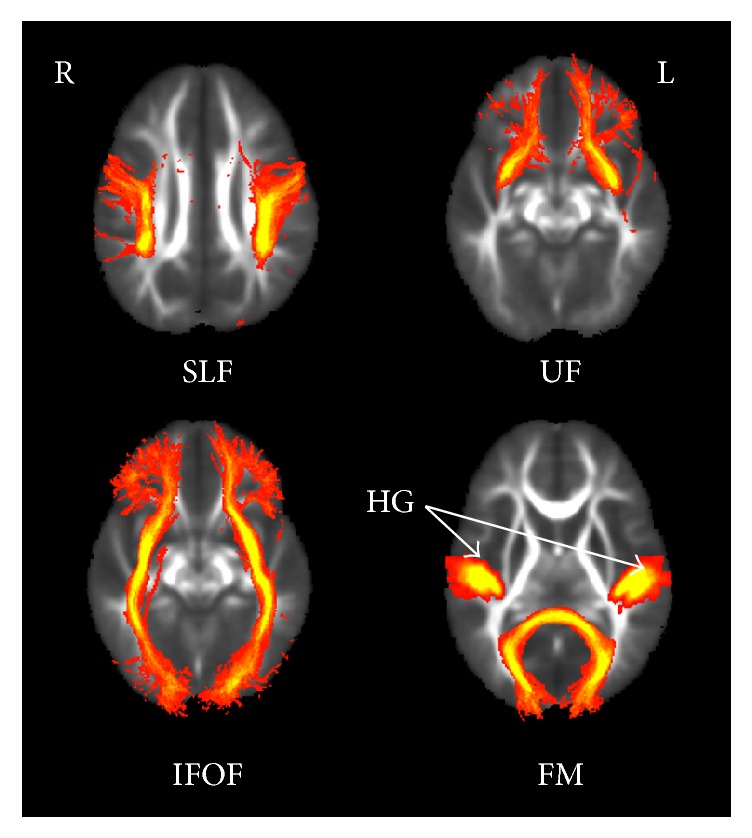
The tracts of interest of the FSL JHU White Matter Tractography Atlas Tool. SLF, superior longitudinal fasciculus; UF, uncinate fasciculus; IFOF, inferior frontooccipital fasciculus; FM, forceps major; HG, Heschl's gyrus.

Voxel-wise statistical analyses were performed across subjects on the skeleton-space FA images. Voxel-wise statistical analysis of individual skeleton images was performed in the randomise package (v2.9) using a nonparametric permutation test. To assess differences between the DEAF and HEAR groups, a null distribution was built up over 5000 permutations on the skeleton mask of projected FA values. The same test was applied after stratification by the age of 4 years. Correlations between FA values and age in each group were also shown on the skeleton mask. To correct for multiple comparisons, we used threshold-free cluster enhancement (TFCE) with the “2D” parameter setting [14]. The significance level was set at *P* < 0.05. We evaluated differences in the mean FA values of each tract of interest, including the superior longitudinal fasciculus (SLF), uncinate fasciculus (UF), inferior frontooccipital fasciculus (IFOF), forceps major (FM), and the white matter tract leading to Heschl's gyrus (HG). The JHU White Matter Tractography Atlas of the FSL atlas tool (http://www.fmrib.ox.ac.uk/fsl/fslview/atlas.html) was applied to the common FA skeleton mask (Figure 1). Individual FA values were extracted by aligning the specific tractography to the FA skeleton mask, and the mean FA values were obtained. The Mann–Whitney *U* test in SPSS ver. 16.0 (SPSS, Chicago, IL, USA) was used to compare between-group differences in the mean FA values for each tract.

## 3. Results

Voxel-wise between-group statistical analyses were performed using skeleton-space fractional anisotropy (FA) images. No significant differences were observed in the FA values within the whole-brain TBSS skeleton between the DEAF and HEAR groups.

**Figure 2:**
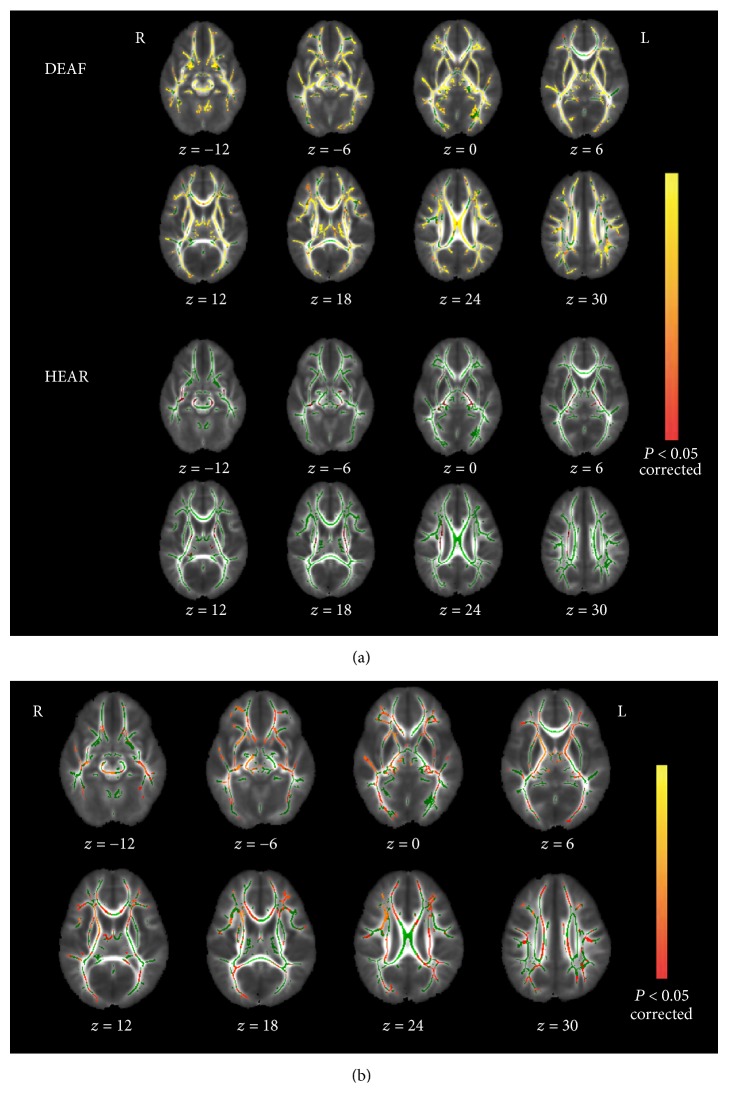
Voxel-wise statistical analyses of skeleton-space FA images. (a) Effects of age on regional FA in both groups. White matter tracts in red-yellow reveal a significant age-related increase in FA. In the DEAF group, significant correlations with age appear for nearly every white matter tract. (b) White matter structures exhibiting significantly lower FAs in the DEAF4 group (*P* < 0.05, corrected for multiple comparisons). The background image is a group-specific brain template. Green voxels are the FA white matter skeleton. Red to yellow voxels show regions of lower FA values in the DEAF4− group compared with the HEAR4− group. Axial sections with *z*-values ranging from −12 to 30 (the MNI coordinates) are shown.

To evaluate correlations between FA values and the ages of each group, we performed voxel-wise correlation analyses of the TBSS skeletons. These revealed a few regions in which the FA values were positively correlated with age in the HEAR group. However, significant positive correlations were evident between FA values and age for almost all white matter tracts in the DEAF group (Figure 2(a)). To further evaluate FA differences between groups, all subjects were stratified by age (cutoff: 4 years). We performed between-group comparisons in subjects younger than 4 years and found significantly lower FA values for many regions of the TBSS skeleton in the DEAF4− group (*n* = 12) compared with the HEAR4− group (*n* = 10) (Figure 2(b)). In subjects older than 4 years, no significant differences were found between groups within the TBSS skeleton.

**Figure 3:**
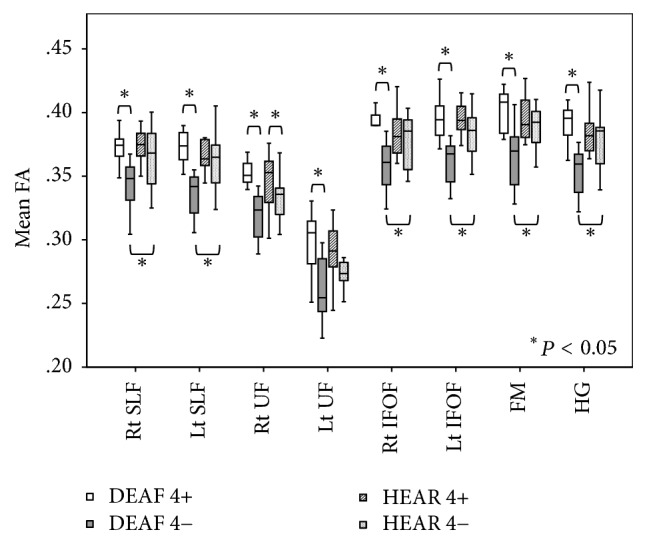
The mean FA values in tracts of interest [the superior longitudinal fasciculus (SLF); the uncinate fasciculus (UF); the inferior frontooccipital fasciculus (IFOF); the forceps major (FM); and the white matter tracts leading to Heschl's gyrus (HG)]. In the DEAF group, DEAF4− subjects (compared with DEAF4+ subjects) exhibited significantly lower FA values in each tract of interest. However, in the HEAR group, no significant difference was apparent between HEAR 4− and HEAR4+ subjects except in the right UF. Of subjects aged <4 years, those in the DEAF4− group exhibited significantly lower FA values in all tracts of interest (except the UF) than did HEAR4− subjects. However, in subjects aged >4 years, no significant difference was apparent between the DEAF4+ and HEAR4+ groups.

We explored differences between the mean FA values of each tract of interest, including the superior longitudinal fasciculus (SLF), uncinate fasciculus (UF), inferior frontooccipital fasciculus (IFOF), forceps major (FM), and the white matter tracts leading to Heschl's gyrus (HG). In the DEAF group, subjects aged <4 years (the DEAF4− group) exhibited significantly lower FA values for each tract compared with the values of those aged >4 years (DEAF4+ group). However, in the HEAR group, we found no significant difference between subjects aged <4 (HEAR 4−) and >4 (HEAR4+) years in any tract of interest, with the exception of the right UF. In subjects aged <4 years, the DEAF4− group exhibited significantly lower FA values in all tracts of interest (except the UF) compared with the HEAR4− group. However, in subjects aged >4 years, no significant differences were evident between the DEAF4+ and HEAR4+ groups (Figure 3).

## 4. Discussion

Recent advances in MRI have facilitated in vivo studies of CNS microstructure [15]. Diffusion tensor imaging (DTI), a form of MRI, reveals the orientation of white matter tracts in vivo and yields a measure of microstructural integrity by quantifying the directionality of water diffusion. In the time since DTI was introduced, many studies have sought correlations between the connectivities of white matter tracts and the pathophysiologies of CNS diseases including multiple sclerosis, Alzheimer's disease, and epilepsy [16].

In the DTI technique, voxel-based morphometry (VBM) and tract-based spatial statistics (TBSS) analysis of diffusion data are the two main methods used to localize white matter changes in a whole-brain manner. VBM is a useful exploratory method and is widely used in anisotropy analysis to detect the changes in white matter which occur in many brain diseases. All of the steps in this method were performed automatically with greater reproducibility than region-of-interest and tractography-based methods. However, the normalization algorithm available in Statistical Parametric Mapping (SPM) software was not designed to handle highly heterogeneous FA images such as children's developing brains, so there is a risk of false-positive results [13]. Another limitation of the VBM method is the arbitrary choice of smoothing kernels. In this study, we used the TBSS technique to overcome the drawbacks of VBM, such as alignment and smoothing issues, because the subjects were young children with developing brains. Additionally, to evaluate differences in the subjects' white matter in the standard brain space, a new target image was chosen: the one with the minimum mean displacement score from all other subjects instead of the standard FMRIB58 FA template used in adult brain studies.

A few studies have used whole-brain DTI analyses to explore white matter changes in prelingually deaf adults and adolescents [17–20]. Kim et al. [17] found that, in deaf patients, FA values were reduced in the internal capsule, the white matter tract lying close to the superior temporal gyrus, the superior longitudinal fasciculus, and the inferior frontooccipital fasciculus. Such changes were interpreted in terms of both disuse-driven atrophy and compensatory plasticity. Recently, Li et al. [19] compared the white matter structures of congenitally deaf individuals, those with acquired deafness, and controls. Deaf individuals exhibited significantly reduced FA values of both superior temporal cortices and the splenium of the corpus callosum compared to hearing-positive controls. A DTI study on prelingually deaf adolescents found that the FA values of the superior temporal gyrus and Heschl's gyrus were lower than normal, consistent with changes reported in adults [20]. Also, deaf adolescents exhibited a significantly reduced auditory brain area volume and/or increased gray matter/WM ratio in Heschl's gyrus and other auditory-related brain areas [18]. However, no whole-brain DTI analyses have yet been performed on prelingually deaf children.

In a DTI study of white matter maturation in early childhood, FA at major neuronal tracts of the brain increased rapidly during the first 12 months but plateaued after 24 months [21]. This increase in FA before 24 months was explained as the influence of the myelination process. Most of the subjects in our study were older than 24 months; we observed a strong correlation between age and FA at almost all white matter tracts in the DEAF group, whereas no correlation appeared for most tracts in the HEAR group. We also observed increased FA with age at several tracts in the DEAF group, based on the extracted regional DTI data. This finding implies that the development of white matter tracts continues until a specific age in deaf children, while it appears to be stable after 2 years of age in normal-hearing children.

Researchers have postulated that the younger deaf children are at the time of CI, the better the outcome of CI is likely to be. Govaerts et al. [22] reported that children who underwent CI before the age of 4 years were likely to score at least 7 on the Categories of Auditory Performance (CAP) scale. Researchers studying the auditory cortex have reported that peak synaptic density is attained at 2–4 years of age in children with normal hearing. After this time, the synaptic counts decrease, and unused synapses are eliminated, reflecting the brain's need to specialize to accommodate prevailing conditions [23]. In congenitally deaf cats, overall synaptic activity was developmentally delayed, with exaggerated synaptic overshoot and increased elimination of synaptic function [24]. Therefore, we performed between-group comparisons after stratification using an age cutoff of 4 years.

In the DEAF4− group, the FA values of many white matter tracts were lower than those in the HEAR4− group. Reduced values of white matter tract FAs in the temporal lobe may be associated with delayed development of such tracts because of loss of auditory input. In contrast, no significant between-group difference was evident in subjects >4 years of age. Moreover, the FA values of the white matter tracts of Heschl's gyrus were significantly correlated with age in the DEAF group, perhaps reflecting cross-modal plasticity, consistent with PET data indicating that under-use of the auditory cortex in prelingually deaf individuals gradually changes over time from a hypometabolic state to a normal or hyperactive presentation [7]. However, our findings do not agree with the results of Miao et al. [20] who studied adolescents. It is difficult to explain the discrepancy, but it is possible that cerebral white matter tract development continues to a certain age in deaf children, and at adolescence, white matter tracts in the temporal lobe may be affected by clinical factors such as learning sign language, visual experiences, and intelligence.

Human language function involves not only the gray matter of circumscribed brain regions in the frontal and temporal cortices but also the white matter fiber tracts connecting these regions [25]. The SLF and UF are white matter tracts connecting Broca's and Wernicke's areas. The SLF is a dorsal pathway from the posterior portion of Broca's area to the superior temporal region and is important in terms of higher-order functionality. The IFOF connects the frontal and occipital lobes and the frontal, posterior parietal, and temporal lobes, and it links the auditory and visual cortices with the prefrontal cortex controlling working memory and executive function [26]. Lee et al. [27] found that deaf children with better executive and visuospatial functions delivered by the prefrontal and parietal cortices were auditorily successful when learning a language after CI. In our present study, the correlations between FA and age in terms of functionality of these white matter tracts were stronger in the DEAF group, reflecting the development of these tracts, which, in turn, affected speech outcomes after CI.

One previous PET study [28] reported that prelingually deaf children aged 5–7 years at CI exhibited the widest variation in individual outcomes, and the child with the broadest hypometabolic area had the best speech perception. They concluded that the extent of hypometabolism as assessed by PET was one of the major predictors of the outcome of CI. We found that the FA values of many white matter tracts were lower in the DEAF4− group than in the HEAR4− group, while no significant differences appeared between groups in older subjects. CI is intended to promote auditory development in children who are deaf; this development will be restricted if cross-modal changes cannot be reversed and normal corticocortical and corticofugal activity is not restored. Therefore, CI should be performed as early as possible to limit the potential for the auditory brain to reorganize. In other words, the auditory brain must be used in early life to ensure that it is not lost to other modalities [29].

## 5. Conclusions

In this study, we used TBSS analysis to investigate WM development in prelingually deaf children. The FA values at many WM tracts were lower in the DEAF group than in the HEAR group younger than 4 years, while no significant differences appeared in older subjects. We also found that the age-related development of WM tracts might continue until 8 years of age in prelingually deaf children. These results are the first to imply the delayed development of cerebral WM tracts in prelingually deaf children, constituting circumstantial evidence that CI should be performed as early as possible to avoid reorganization of the auditory brain.
